# Dental Status is Associated With Incident Functional Disability in Community-Dwelling Older Japanese: A Prospective Cohort Study Using Propensity Score Matching

**DOI:** 10.2188/jea.JE20180203

**Published:** 2020-02-05

**Authors:** Takamasa Komiyama, Takashi Ohi, Yasutake Tomata, Fumiya Tanji, Ichiro Tsuji, Makoto Watanabe, Yoshinori Hattori

**Affiliations:** 1 Division of Aging and Geriatric Dentistry, Department of Oral Function and Morphology, Tohoku University Graduate School of Dentistry, Sendai, Japan; 2 Japanese Red Cross Ishinomaki Hospital, Miyagi, Japan; 3 Division of Epidemiology, Department of Health Informatics and Public Health, Tohoku University School of Public Health, Graduate School of Medicine, Sendai, Japan; 4 Department of Social Welfare, Faculty of General Welfare, Tohoku Fukushi University, Sendai, Japan

**Keywords:** prospective cohort study, oral health, functional disability, propensity score matching

## Abstract

**Background:**

A growing number of epidemiology studies have shown that poor oral health is associated with an increased incidence of functional disability. However, there are few studies in which the confounding bias is adjusted appropriately. In this study, we examined whether dental status is associated with functional disability in elderly Japanese using a 13-year prospective cohort study after elimination of confounding factors with propensity score matching.

**Methods:**

Participants were community-dwelling Japanese aged 70 years or older who lived in the Tsurugaya district of Sendai (*n* = 838). The number of remaining teeth (over 20 teeth vs 0–19 teeth) was defined as the exposure variable. The outcome was the incidence of functional disability, defined as the first certification of long-term care insurance (LTCI) in Japan. The variables that were used to determine propensity score matching were age, sex, body mass index (BMI), medical history (stroke, hypertension, myocardial infarction, cancer, and diabetes), smoking, alcohol consumption, educational attainment, depression symptoms, cognitive impairment, physical function, social support, and marital status.

**Results:**

As a result of the propensity score matching, 574 participants were selected. Participants with 0–19 teeth were more likely to develop functional disability than those with 20 or more teeth (hazard ratio 1.33; 95% confidence interval, 1.01–1.75).

**Conclusions:**

In this prospective cohort study targeting community-dwelling older adults in Japan, having less than 20 teeth was confirmed to be an independent risk factor for functional disability even after conducting propensity score matching. This study supports previous publications showing that oral health is associated with functional disability.

## INTRODUCTION

Although the increase in the number of older adults and the rise in aging rates have been rapidly progressing all over the world, these phenomena have been more pronounced in Japan than in other countries.^1^ In planning of comprehensive policies for the aging population, it is generally accepted that an extension of the healthy life expectancy, which delays the incidence of functional disabilities in the elderly, is one of the important measures. As one of the measures to extend the healthy life expectancy, a satisfactory nutritional status and the maintenance of oral health, which is closely related to eating, have been identified.^2^

A growing number of epidemiological studies have shown that oral health is associated with systemic diseases, such as dementia and stroke,^3^^,^^4^ as well as with disability, reflected by a decline in physical functions and an increased incidence of falls.^5^^,^^6^ Furthermore, several studies have demonstrated the relationship between oral health status and incidence of functional disability, which is a major indicator of healthy life expectancy. However, in many studies, the influence of confounding bias was not fully considered. In a 4-year prospective cohort study that examined the relationship between the number of teeth and the incidence of functional disability, cognitive function was not treated as a confounding factor.^7^ In a 6-year prospective cohort study that investigated the relationship between the dentition status and the incidence of functional disability, socioeconomic status was not considered as a confounding factor.^8^ In prospective cohort studies that examined the relationship between the incidence of functional disability and the number of teeth or the occlusal force, the influence of confounding variables has probably not been sufficiently eliminated, since a conventional multivariate analysis was performed.^5^^,^^9^^,^^10^ A previous study actually pointed out that there are many confounding factors in the relationship between oral health status and functional disability; hence, it is necessary to adequately consider the influence of confounding bias.^7^

Propensity score matching is a statistical method that can reduce the influence of confounding bias as much as possible and has been applied in many epidemiological studies.^2^^,^^11^^,^^12^ However, only a limited number of oral epidemiological studies have applied propensity scores. Some reports have examined the relationship between tooth loss and mortality using propensity scores as a covariate.^13^^,^^14^

In this prospective cohort study, we focused on the number of remaining teeth, which is a representative indicator of oral health, and conducted propensity score matching to reduce the influence of confounding bias as much as possible. With this dataset, we investigated the relationship between the number of remaining teeth and the incidence of functional disability in community-dwelling older adults in Japan.

## METHODS

### Study design

This study was conducted as a part of the comprehensive geriatric assessment (CGA), the “Tsurugaya project”, which enrolled elderly people aged 70 years or older residing in the Tsurugaya district of Sendai in northern Japan. In a baseline survey in 2003, the participants were examined for their number of teeth. Information about the incidence of functional disability, defined as the first certification of long-term care insurance (LTCI), was collected through June 2016.

### Study participants

**Figure 1.**
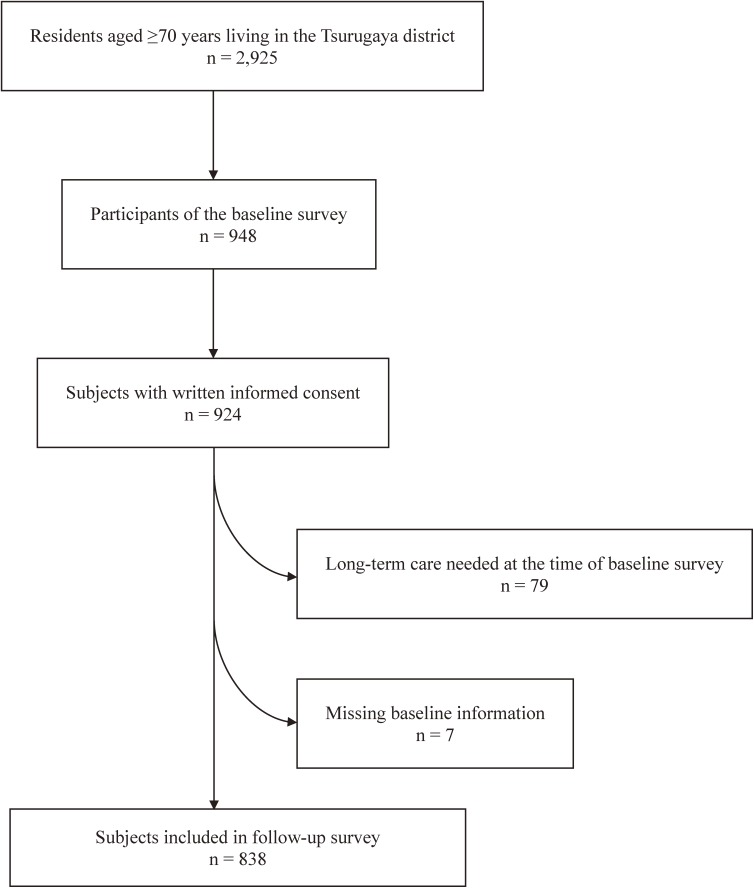
Flow diagram of study participants

A flow diagram of the study participants is shown in Figure 1. Study concept, design, and details of the “Tsurugaya project” have been described in previous publications.^15^^,^^16^ Briefly, in 2003, the “Tsurugaya project” aimed to identify risk factors for the occurrence of functional disability in community-dwelling elderly adults by implementing the CGA. We invited all residents of the Tsurugaya district with an age ≥70 years to participate in the CGA. Of those invited, 948 took part in the baseline survey (response rate, 32.4%). Of these respondents, 924 provided written informed consent for access to LTCI information and the associated data analysis. We excluded 79 participants from a follow-up survey because of certified LTCI (ie, a functional disability in the baseline survey) and 7 participants because of missing data regarding their number of remaining teeth. Ultimately, 838 participants were included in the final analysis (mean age, 75.2 [standard deviation, 4.5] years; male, 48.2%). All participants were asked to read and sign a detailed informed consent document. The institutional review board of Tohoku University Graduate School of Medicine approved the study protocol.

### Oral health measurements

Oral health was evaluated as a part of the CGA.^17^ Five specially trained dentists counted the number of remaining teeth. Any retained roots were excluded from this number. Using a cutoff value of 20 teeth, the participants were divided into two groups for further analyses.^7^^,^^10^^,^^14^^,^^18^

### Additional measurements

The participants were surveyed regarding age, sex, body mass index (BMI), medical history (stroke, hypertension, myocardial infarction, cancer, and diabetes), smoking, alcohol consumption, duration of education, depressive symptoms, cognitive function, physical function, social support, and marital status. Age and BMI were treated as continuous variables. Medical history, smoking, alcohol consumption, duration of education, and marital status were determined using a questionnaire. Smoking status was classified as current smoking, past smoking, or non-smoking. Similarly, alcohol consumption was divided into current alcohol consumption, past alcohol consumption, or non-alcohol consumption. We treated educational attainment as socioeconomic status and classified age upon graduation from last school as less than 18 years and 18 years or older. Marital status was classified as married, widowed or divorced, or never married. We coded the marital status as married or other. Depressive symptoms were surveyed using the Japanese version of the 30-item Geriatric Depression Scale (GDS),^19^ with scores ≥11 indicating depressive symptoms. Cognitive function was measured using the Mini-Mental State Examination (MMSE), with scores ≥26 indicating normal cognition and scores <26 indicating cognitive impairment.^20^ Physical function was assessed using the 6-item physical function scale of the Short-Form General Health Survey adapted from the Medical Outcome Study (MOS),^21^ with scores of 0–4 indicating moderate or low physical activity. The presence of social support was evaluated using the following questions: Do you have someone [1] with whom you can consult when you are in trouble? [2] with whom you can consult when your physical condition is poor? [3] who can help with your daily housework? [4] who can take you to a hospital when your physical condition is not good? and [5] who can take care of you when you are ill in bed? If participants did not respond with “yes” to all questions, they were classified as having restricted social support.^17^

### Follow-up and outcome

The primary outcome of the present study was the incidence of functional disability, defined as the first certification of LTCI, which in Japan is strictly and widely utilized as a national care insurance system. Several previous epidemiological studies have adopted this criterion as the primary outcome.^7^^,^^22^^,^^23^ The details of the LTCI system in Japan have been described in previous studies.^24^ Briefly, subjects over 65 years of age with functional disability are eligible for LTCI services. In order to receive LTCI, the recipients themselves, their family, or their care manager must apply for it. For all subjects to receive LTCI, the municipality recommends an application to the subjects or they act on behalf of the applicant. Therefore, it is considered that most functionally disabled people receive support through the LTCI system.^25^ After the application, the first assessment is made by physicians after consultation with the municipality staff and application of the questionnaire, which determines physical and mental functions. Based on this assessment, if the applicant is judged to be certificated, the Municipal Certification Committee attributes the applicant to one of seven levels of care and support.

Information regarding the LTCI certification (date and level), death, and relocation outside Sendai was obtained from the Sendai Municipal Authority. The follow-up time was from the baseline survey in 2003 until the end of June 2016. A censored case was defined as relocation outside Sendai or death.

### Propensity score matching

In order to reduce the influence of confounding factors, we divided the participants into two groups of 20 or more teeth and fewer than 20 teeth and carried out a greedy propensity score matching at a 1:1 ratio. According to the previous studies, the variables used for this procedure were age, sex, BMI, medical history (stroke, hypertension, myocardial infarction, cancer, and diabetes), smoking, alcohol consumption, educational attainment, depressive symptoms, cognitive function, physical function, social support, and marital status.^7^^,^^9^ We confirmed multicollinearity of all the variables about the propensity score matching using the variance inflation factor (VIF). On the logit of the propensity score, we employed caliper widths equal to 0.2 of the standard deviation.^11^

The propensity scores of the 20 or more teeth group and the fewer than 20 teeth group were compared. C-index was evaluated to confirm good discrimination. Hosmer-Lemeshow test was evaluated to confirm goodness of fit. Standardized differences were determined for all baseline variables before and after matching to evaluate the imbalance before and after matching. A standardized difference of less than 10% for a given variable indicated a relatively small imbalance.^26^

### Statistical analysis

The baseline characteristics before and after matching according to the number of teeth were evaluated using standardized differences. The relationship between the number of teeth and the incidence rate of functional disability was assessed using Kaplan-Meier survival curves and log-rank tests. The hazard ratios (HRs) and 95% confidence intervals (CIs) were calculated using Cox proportional hazards model, which examined the relationship between the number of teeth and the relative risk of functional disability.

Because categorical data, which included missing data, were created with “missing” categories, participants with “missing” information were included in the analysis to maximize the statistical power.

We performed two sensitivity analyses. To disprove a reversal of the causal relationship, one of the analyses excluded those participants whose functional disability occurred within 6 months after the baseline survey. The other analysis used the propensity score as a covariate.

JMP software v13 (SAS Institute, Inc., Cary, NC, USA) was used for the calculation of propensity scores and matching; all other analyses were performed with SPSS v21 (IBM Software Group, Chicago, IL, USA). All analyses were carried out as two-tailed tests and were considered to be significant if *P*-values were less than 0.05.

## RESULTS

**Figure 2.**
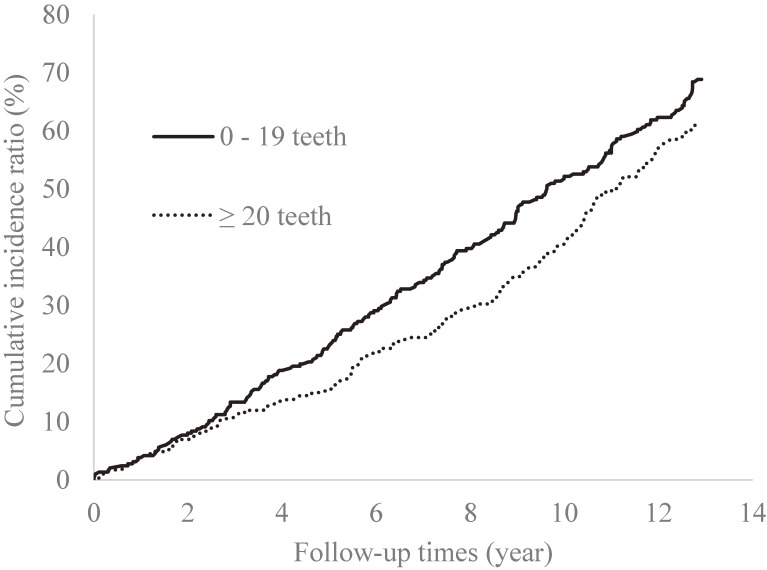
Kaplan-Meier curves showing the cumulative incidences of functional disability according to the number of remaining teeth after propensity score matching. Log-rank test, *P* = 0.03

In total, we observed 6,870 person-years with an average of 8.2 years and a maximum of 13 years. An LTCI first certification was received by 518 participants, 76 participants died, and 17 participants relocated outside Sendai area. Examining the multicollinearity of the variables used for the propensity score using VIF, all variables of VIF were less than 10 (data not shown). According to propensity score matching, 287 participants were selected for each of the two groups defined by the cutoff value of 20 teeth (574 participants in total). In this propensity score model, goodness of fit was secured (Hosmer-Lemeshow test; *P* = 0.78) and c-index was 0.72. Participants who were selected for matching were younger, had lower ratings for cognitive and physical functions, with a higher proportion of subjects with current alcohol consumption than those not selected (data not shown). Of the participants selected via propensity score matching, there were 341 participants for the first certification of LTCI, 49 participants for death, and 12 participants for relocation outside Sendai. Table 1 shows the baseline characteristics of participants according to their number of teeth before and after the matching procedure. After propensity matching, the standardized differences of all variables were less than 10%. Figure 2 demonstrates that the cumulative incidence of functional disability was significantly higher in the matched group with fewer than 20 teeth (log-rank test; *P* = 0.03). This group also had a significantly higher HR (HR 1.33; 95% CI, 1.01–1.75; Table 2) calculated according to the Cox proportional hazards model.

**Table 1.** Baseline characteristics before and after propensity score matching according to the number of remaining teeth

	Before matching			After matching		

	≥20 teeth (*n* = 374)	0–19 teeth (*n* = 464)	Standardized difference (%)	≥20 teeth (*n* = 287)	0–19 teeth (*n* = 287)	Standardized difference (%)
Age, mean (SD)	73.7 (3.4)	76.4 (4.8)	14.7	74.2 (3.6)	74.3 (3.3)	0.8
Male, %	45.0	52.1	14.2	48.4	48.1	0.6
BMI, mean (SD)	24.4 (3.1)	23.9 (3.5)	4.4	24.3 (3.2)	24.1 (3.7)	1.5
Stroke, %	1.9	4.5	14.8	2.4	3.1	4.3
Hypertension, %	44.7	38.6	12.4	41.5	41.1	0.8
Myocardial infarction, %	11.5	9.3	7.2	10.5	11.5	3.5
Cancer, %	8.0	9.7	6.0	9.4	9.4	0
Diabetes, %	15.0	14.9	0.3	14.3	14.6	0.9
Current smoker, %	7.2	12.7	18.5	9.1	10.5	4.4
Alcohol consumption, %	50.3	37.1	26.9	44.6	46.3	3.4
Age upon graduation from last school <18 years, %	29.9	35.8	12.6	32.8	34.2	3.0
Depressive symptoms, %	21.4	27.4	14.0	23.3	22.7	1.7
Cognitive impairment, %	6.4	11.2	17.0	7.3	7.0	1.2
Vigorous physical function, %	21.1	27.8	15.6	22.3	22.7	0.7
Lack of social support, %	32.4	34.5	4.5	32.8	35.2	5.3
Married, %	75.4	62.1	29.0	71.1	69.7	3.1

SD, standard deviation.

The numbers of subjects with available data for smoking status, alcohol consumption, educational attainment, depressive symptoms, cognitive impairment, physical function, and marital status were 825, 762, 821, 831, 819, 836, and 837, respectively.

**Table 2.** The relationship between the number of remaining teeth and the incidence of functional disability (Cox proportional hazards model after propensity score matching)

	HR (95% CI)		*P*-value

	≥20 teeth (*n* = 287)	0–19 teeth (*n* = 287)	
Person-years	2,576	2,360	
Incidents, *n*	160	181	
Incidents/1,000 person-years	62.1	76.7	
After propensity score matching model	1.00 (reference)	1.33 (1.01–1.75)	0.04

CI, confidence interval; HR, hazard ratio.

In the sensitivity analysis that excluded participants with a functional disability within 6 months after the baseline survey, after propensity matching, the standardized differences of all variables were less than 10% (eTable 1), and the group with fewer than 20 teeth remained at increased risk (HR 1.32; 95% CI, 1.00–1.73) (eTable 2). Similarly, in the second sensitivity analysis, which used the propensity score as a covariate, the HR of functional disability was significantly higher in the group with fewer than 20 teeth (eTable 3). The relative risk and the cumulative incidence of functional disability were also significantly higher in the group with fewer than 20 teeth when we applied a conventional multivariate Cox proportional hazards model that did not use propensity scores (eFigure 1 and eTable 4).

## DISCUSSION

In this prospective cohort study with an average of 8.2 years, we removed the influence of confounding bias as much as possible to investigate the relationship between the number of remaining teeth and incidence of functional disability. The data revealed that having less than 20 teeth is associated with an increased incidence of functional disability in community-dwelling older adults in Japan. This is, to the best of our knowledge, the first study that examines this relationship using propensity score matching. Thus, our study confirms that poor dental status is an independent risk factor for functional disability.

There are several possible mechanisms by which poor dental status may be related to an earlier onset of functional disability in an elderly community-dwelling Japanese population. Tooth loss, which might lead to a chewing dysfunction, is related to changes in nutrition intake and malnutrition.^27^^–^^30^ It has been reported that changes in nutrition intake and malnutrition are risk factors for dementia, stroke, and frailty.^31^^–^^33^ These diseases and systemic conditions were the major cause of LTCI certifications, which was the primary outcome in the present study (stroke, 18.5%; dementia, 15.8%; frailty, 13.4%).^34^

Several previous studies have reported a correlation between oral health status and incidence of functional disability. In a 4-year cohort study following 4,425 community-dwelling older adults, it was reported that the relative risk of functional disability in the group with fewer than 20 teeth was 1.21 (95% CI, 1.06–1.40) in comparison to the group with 20 teeth or more.^7^ In a 10-year cohort study following 460 community-dwelling adults aged 70 years or more, it was reported that the presence of fewer than 20 teeth was an independent risk factor for functional disability.^35^ A 12-year cohort study following 3,166 subjects aged 60 years or older demonstrated that edentulism is an independent risk factor for functional disability.^5^ The present study is consistent with these previous publications and supports their results that show the significant relationship between oral health status and incidence of functional disability.

In the present study, we performed the sensitivity analyses. First, to disprove a reversal of the causal relationship, excluding the participants with a functional disability within 6 months after the baseline survey, the HR and 95% CI of the group with fewer than 20 teeth was confirmed (HR 1.32; 95% CI, 1.00–1.73). We confirmed that the number of remaining teeth was related to subsequent incident functional disability even after reducing the possibility of reverse causal effect. Second, we examined the relationship between the number of remaining teeth and incidence of functional disability using the propensity score as a covariate. In addition, we calculated the HR and 95% CI using the conventional Cox proportional analysis without using the propensity score. Both results were similar to the results of examining the relationship between the number of remaining and incidence of functional disability using propensity score matching. It was confirmed that the relationship between the number of remaining teeth and functional disability did not change even after using similar statistical analysis and this relationship was not frail.

In the present study, we focused on evaluating the oral health status with a 20-teeth cutoff. It has been shown that subjects with 20 teeth or more have better masticatory function than those with less than 20 teeth.^36^ In addition, this indicator has been used by many epidemiological studies and has been employed as a risk factor for physical function, cognitive function, and mortality that is associated with functional disability. The concept of shortened dental arch also supports the idea that the classification of dental status is based on 20 teeth.^37^ Furthermore, the World Health Organization, considering tooth loss as a global issue and public health problem, states that retention of 20 teeth or more is desirable in the elderly individuals.^38^ The present study showed that the number of remaining teeth with a cut-off 20 was associated with functional disability.

The present study has several limitations. First, there is selection bias in the present study. Since the present study was conducted as a part of the CGA, it is considered that healthier or more health conscious participants attended the baseline survey (response rate: 32.4%). A cohort study focusing on oral health status targeted community-dwelling older adults in the same period and achieved a response rate of 72.9%. In the previous study, the rate of those with 20 teeth or more was 28.4%, while in the present study the same parameter was 44.6%.^39^ In addition, the participants selected for propensity score matching were healthier than the participants not selected for propensity score matching. Although these selection biases need to be considered when generalizing the results of the present study, they does not affect the relationship between the number of remaining teeth and incidence of functional disability; that is, they do not affect the internal validity of the present study. In addition, if elderly subjects with poor oral health did not participate appropriately in the present study, it can be expected that the relationship between the number of remaining teeth and incidence of functional disability is rather underestimated. Second, in the present study, the cause of the LTCI certification was not investigated. Since the causes of LTCI are diverse, it could not be clarified how poor dental status was related to the occurrence of functional disability. In future studies, the cause of LTCI certifications should be examined to elucidate the mechanisms by which poor dental status leads to an increased incidence of functional disability. Third, we divided the number of remaining teeth into two groups with the cut-off value of 20. Generally, oral function of older adults who have fewer remaining teeth is affected by receiving prosthodontic treatment. Since the participants of the present study were healthier older adults, only 2.7% of participants with less than 20 teeth did not receive prosthodontic treatment. Although we examined the relationship between number of teeth and incidence of functional disability excluding the participants with less than 20 teeth who did not receive prosthodontic treatment, the results did not conflict with the observed relationship between number of remaining teeth and functional disability (data not shown). In the present study, although the presence of prosthodontic treatment was surveyed, we did not evaluate the detailed effect of prosthodontic treatment. If evaluating the detailed effect of prosthodontic treatment, especially targeting to older adults with fewer remaining teeth, it is expected to elucidate the pathway by which poor dental status causes incidence of functional disability.

### Conclusions

The findings of this prospective cohort study in propensity score-matched community-dwelling Japanese adults aged 70 years or more indicate that poor dental status can be considered to be an independent risk factor for an increased incidence of functional disability. The results of the present study support the findings of previous studies showing this relationship between oral health and incidence of functional disability. In order to further investigate this relationship, it is desirable to carry out systematic reviews or meta-analyses.

## APPENDIX A. SUPPLEMENTARY DATA

eFigure 1. Kaplan-Meier curves showing the cumulative incidences of functional disability according to the number of remaining teeth before propensity score matching. Log-rank test, *P* < 0.005

eTable 1. Baseline characteristics before and after propensity score matching according to the number of remaining teeth (excluding incidences of functional disability within the first 6 months)

eTable 2. The relationship between the number of remaining teeth and the incidence of functional disability excluding incidences of functional disability within the first 6 months (Cox proportional hazards model after propensity score matching)

eTable 3. The relationship between the number of remaining teeth and the incidence of functional disability (Cox proportional hazards model using the propensity score as a covariate)

eTable 4. The relationship between the number of remaining teeth and the incidence of functional disability (Cox proportional hazards model before propensity score matching)
