# Supplementary material for: Dental Status is Associated With Incident Functional Disability in Community-Dwelling Older Japanese: A Prospective Cohort Study Using Propensity Score Matching
Source: J Epidemiol. 2020 Feb 5;30(2):84–90. doi: 10.2188/jea.JE20180203 (PMC6949184; doi:10.2188/jea.JE20180203)
Supplement: Supplementary file 1 [file je-30-084-s001.pdf]

Dental Status Is Associated with Incident Functional Disability in Community-Dwelling Older Japanese: A Prospective Cohort Study Using Propensity Score Matching

eTable 1. Baseline characteristics before and after propensity score matching according to the number of remaining teeth (excluding incidences of functional disability within the first 6 months)

	Before matching			After matching		
	≥20 teeth (n=366)	0–19 teeth (n=450)	Standardized difference (%)	≥20 teeth (n=284)	0–19 teeth (n=284)	Standardized difference (%)
Age, mean (SD)	73.6 (3.4)	76.2 (4.7)	14.8	74.2 (3.6)	74.2 (3.4)	0
Male, %	52.5	45.3	14.4	48.2	48.2	0
BMI, mean (SD)	24.4 (3.1)	24.0 (3.5)	3.6	24.3 (3.2)	24.1 (3.6)	1.7
Stroke, %	1.9	4.4	14.4	2.5	2.8	1.9
Hypertension, %	44.3	38.7	11.4	40.9	42.6	3.6
Myocardial infarction, %	10.9	8.9	6.7	11.3	9.5	5.9
Cancer, %	7.7	9.8	7.8	9.2	8.8	1.0
Diabetes, %	15.0	15.1	0.3	15.1	15.9	1.9
Current smoker, %	7.4	13.1	18.9	8.8	9.2	1.0
Alcohol consumption, %	50.3	37.3	26.4	43.3	44.0	1.4
Age upon graduation from last school <18 years, %	30.3	35.1	10.2	32.4	33.5	2.1
Depressive symptoms, %	21.3	26.7	12.7	22.5	23.9	3.3
Cognitive impairment, %	6.6	11.3	16.5	7.0	9.9	9.9

Vigorous physical function, %	19.7	27.8	19.1	22.2	22.5	0.7
Lack of social support, %	32.2	33.1	1.9	32.8	33.5	1.5
Married, %	75.1	62.7	27.0	71.1	71.5	0.9

BMI, body mass index; SD, standard deviation.

eTable 2. The relationship between the number of remaining teeth and the incidence of functional disability excluding incidences of functional disability within the first 6 months (Cox proportional hazards model after propensity score matching)

	HR (95% CI)		p-value
	≥20 teeth (n=284)	0–19 teeth (n=284)	
Person-years	2,594	2,389	
Incidents, n	153	171	
Incidents/1,000 person-years	59.0	71.6	
After propensity score matching model	1.00 (reference)	1.32 (1.00–1.73)	0.05

CI, confidence interval; HR, hazard ratio.

eTable 3. The relationship between the number of remaining teeth and the incidence of functional disability (Cox proportional hazards model using the propensity score as a covariate)

	HR (95% CI)		p-value
	≥20 teeth (n=374)	0–19 teeth (n=464)	
Person-years	3,494	3,375	
Incidents, n	192	326	
Incidents/1,000 person-years	55.0	96.6	
After propensity score matching model	1.00 (reference)	1.27 (1.04-1.53)	0.02

CI, confidence interval; HR, hazard ratio.

eTable 4. The relationship between the number of remaining teeth and the incidence of functional disability (Cox proportional hazards model before propensity score matching)

					HR (95% CI)		
	Participants, n	Person-years	Incidents, n	Incidents/1,000 person-years	Model 1[a]	Model 2[b]	Model 3[c]
Number of remaining teeth							
≥20 teeth	374	3,495	192	54.9	1.00 (reference)	1.00 (reference)	1.00 (reference)
0–19 teeth	464	3,375	326	96.5	1.92 (1.60–2.30) $p<0.001$	1.44 (1.19–1.73) $p<0.001$	1.32 (1.09–1.60) $p=0.005$

CI, confidence interval; HR, hazard ratio.

[a]Model 1: Unadjusted.

[b]Model 2: Adjusted for age and sex.

[c]Model 3: Adjusted for age, sex, body mass index, medical history, smoking, alcohol consumption, educational attainment, depressive symptoms, cognitive impairment, physical function, social support, and marital status.

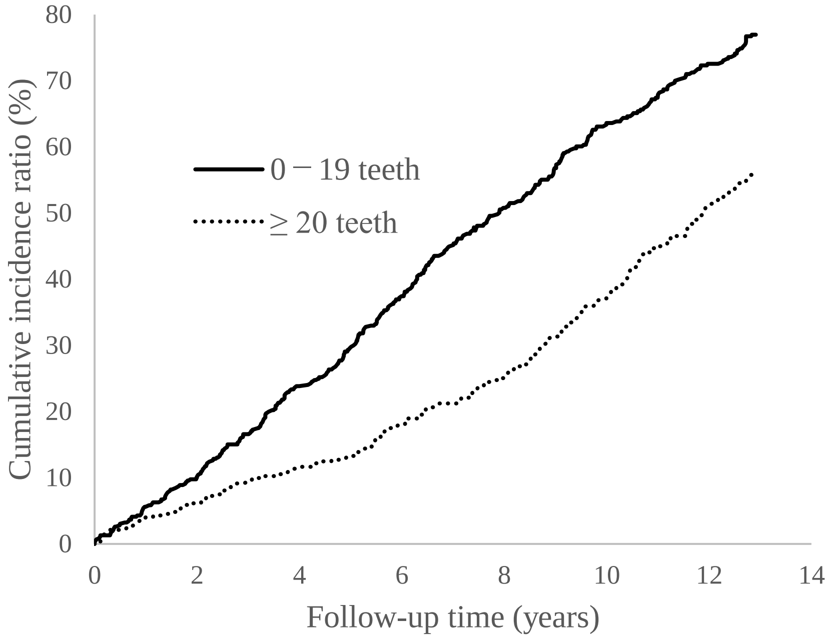

eFigure 1. Kaplan-Meier curves showing the cumulative incidences of functional disability according to the number of remaining teeth before propensity score matching. Log-rank test, $p<0.005$
